# Comparison of the efficacy and complications of tolterodine and α-adrenergic receptor blockers in improving ureteral stent-related symptoms: A systematic review and meta-analysis

**DOI:** 10.1371/journal.pone.0302716

**Published:** 2024-05-03

**Authors:** Ming Liu, Shangjing Liu, Qiancheng Mao, Qingsong Zou, Yuanshan Cui, Jitao Wu

**Affiliations:** 1 Second Clinical Medical College, Binzhou Medical University, Yantai, Shandong, China; 2 Department of Urology, Yantai Yuhuangding Hospital, Qingdao University, Yantai, Shandong, China; Faculty of Medicine, Saint-Joseph University, LEBANON

## Abstract

**Objective:**

We conducted a systematic evaluation of the therapeutic efficacy and complications of tolterodine and α-adrenergic receptor blockers in alleviating ureteral stent-related symptoms.

**Methods:**

Until August 2023, we conducted a comprehensive literature search on PubMed, Embase, Web of Science, and Cochrane Library to identify randomized controlled trials evaluating the efficacy and complications of tolterodine and α-adrenergic receptor blockers in treating ureteral stent-related symptoms. Two reviewers independently screened studies and extracted data. The scores from various domains of the Ureteral Stent Symptom Questionnaire (USSQ) were summarized and compared, and statistical analysis was performed using RevMan 5.4.0 software.

**Results:**

A total of 8 studies met the inclusion criteria for our analysis. These studies were conducted at different centers. All studies were randomized controlled trials, involving a total of 487 patients, with 244 patients receiving α-adrenergic receptor blockers and 243 patients receiving tolterodine. The results showed that tolterodine demonstrated significantly better improvement in body pain (MD, 1.56; 95% CI [0.46, 2.66]; p = 0.005) (MD, 0.46; 95% CI [0.12, 0.80]; p = 0.008) (MD, 3.21; 95% CI [1.89, 4.52]; p = 0.00001) among patients after ureteral stent placement compared to α-adrenergic receptor blockers at different time points. Additionally, at 4 weeks, tolterodine showed superior improvement in general health (MD, 0.15; 95% CI [0.03, 0.27]; p = 0.01) and urinary symptoms (MD, 1.62; 95% CI [0.59, 2.66]; p = 0.002) compared to α-adrenergic receptor blockers, while at 6 weeks, tolterodine showed better improvement in work performance (MD, -1.60; 95% CI [-2.73, -0.48]; p = 0.005) compared to α-adrenergic receptor blockers. Additionally, the incidence of dry mouth (RR, 4.21; 95% CI [1.38, 12.87]; p = 0.01) is higher with the use of tolterodine compared to α-adrenergic receptor blockers. However, there were no significant statistical differences between the two drugs in other outcomes.

**Conclusion:**

This meta-analysis suggests that tolterodine is superior to α-adrenergic receptor blockers in improving physical pain symptoms after ureteral stent placement, while α-adrenergic receptor blockers are more effective than tolterodine in enhancing work performance. Additionally, the incidence of dry mouth is higher with the use of tolterodine compared to α-adrenergic receptor blockers. However, higher-quality randomized controlled trials are needed to further investigate this issue.

## Introduction

The ureteral stent, an indispensable tool in urological surgery, plays a paramount role in the management of conditions such as ureteral stones, renal decompression, obstruction prevention, and facilitation of tissue repair [1]. However, the placement of a ureteral stent gives rise to a range of stent-related symptoms, including lower urinary tract symptoms, hematuria, pain, and infection, which prove arduous to ameliorate and significantly impair the quality of life for patients [2]. The precise pathophysiological mechanisms underlying these symptoms remain elusive, with the prevailing belief attributing them to the mechanical irritation of the bladder trigone by the ureteral stent, ureteral reflux, and the physical properties of the stent itself. To alleviate the stent-related symptoms, clinical approaches commonly encompass pharmacotherapy, stent modification, and pharmacological removal of the stent [3, 4]. Despite endeavors to optimize the physical characteristics of ureteral stents, such as material composition, shape, and dimensions, the outcomes have regrettably fallen short of satisfaction.

Antimuscarinics and α-adrenergic receptor blockers are first-line medications for treating lower urinary tract symptoms (LUTS) in patients with benign prostatic hyperplasia (BPH) and overactive bladder (OAB). Since the urinary symptoms caused by ureteral stents are similar to those caused by OAB and BPH, studies have explored the use of α-adrenergic receptor blockers and antimuscarinics to treat ureteral stent-related symptoms [5, 6]. Currently, the main α-adrenergic receptor blockers used to alleviate discomfort associated with ureteral stents include tamsulosin, alfuzosin, and terazosin. Tolterodine, a competitive antimuscarinic, has demonstrated good tolerability and clinical efficacy in improving lower urinary tract symptoms in patients with OAB [7]. Research has also shown that the use of tolterodine can significantly improve urinary symptoms in patients following ureteral stent placement [8].

Recently, several randomized controlled studies evaluating the efficacy of tolterodine and α-adrenergic receptor blockers in treating ureteral stent-related symptoms have been published. However, these studies have not reached a consistent conclusion, and there has been no unified comparison and evaluation of the efficacy between tolterodine and α-adrenergic receptor blockers. As there is currently no systematic review and analysis available, we have collected studies that meet the criteria and systematically evaluated the efficacy and safety of tolterodine or α-adrenergic receptor blockers as monotherapy for treating symptoms related to ureteral stents.

## Methods

### Search strategy and inclusion criteria

Our study rigorously adhered to the Preferred Reporting Items for Systematic Reviews and Meta-analyses (PRISMA) guidelines [9]. Before August 2023, a meticulous and comprehensive literature search was conducted on esteemed online databases such as PubMed, Web of Science, Embase, ResearchGate, and the Cochrane Library to ascertain randomized controlled trials (RCTs). The search strategy employed is outlined below: (tolterodine) AND (ureteral stent-related symptoms OR ureteric stent-related discomfort OR SRS). Furthermore, a manual examination of the references and relevant articles from clinical studies and reviews was conducted. The language was restricted to English.

### The inclusion criteria were studies that

- The patient underwent a ureteral stent implantation procedure.
- The efficacy of tolterodine compared to α-adrenergic receptor blockers in treating ureteral stent-related symptoms was compared.
- Full-text and analyzable data were available.
- For the randomized controlled trial.

**Fig 1:**
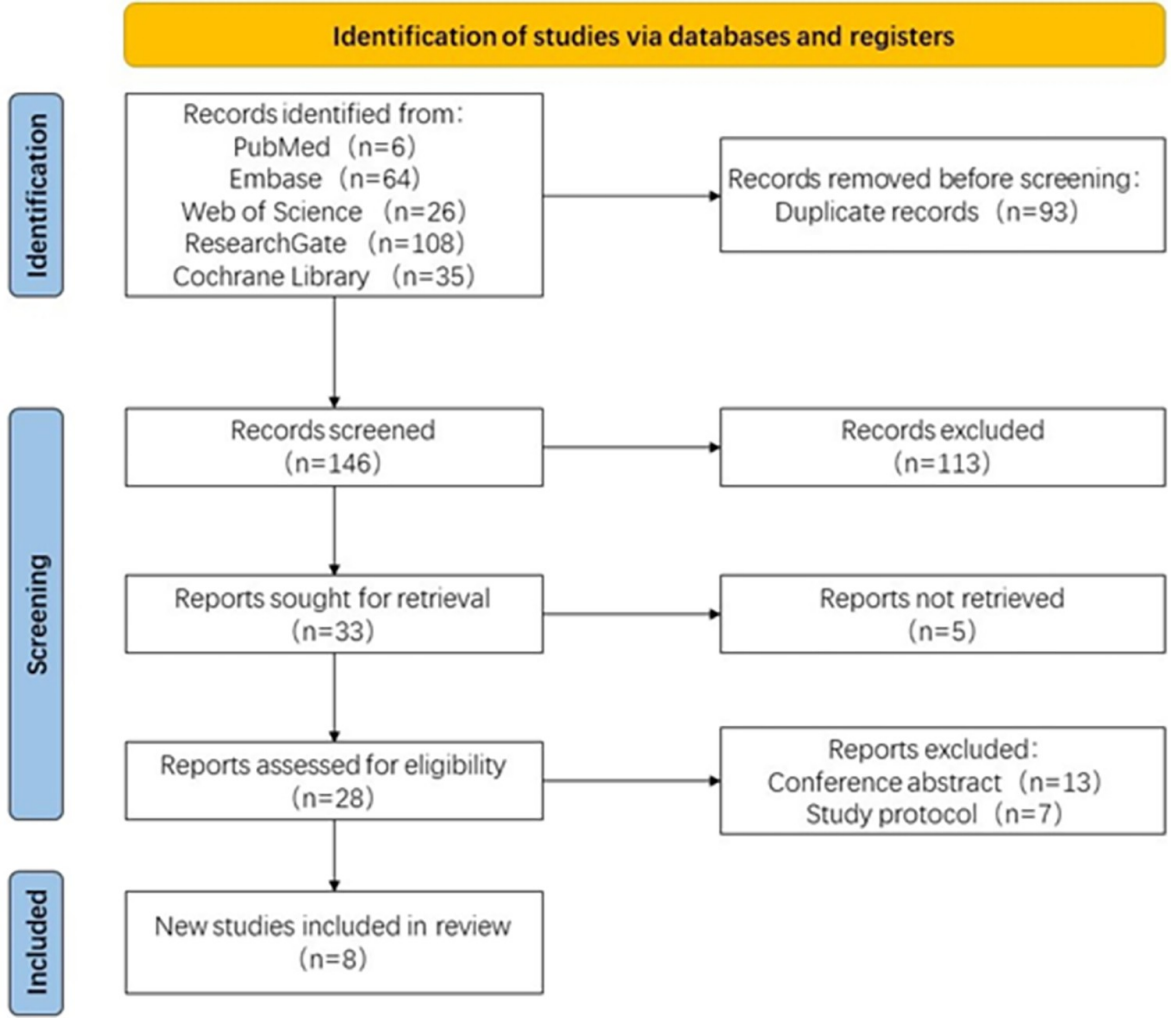
Flow diagram of the study selection process.

Three authors independently reviewed all the retrieved articles and reached a consensus through discussion to determine the final inclusion of articles. The flowchart depicting this process is shown in **Fig 1**.

### Quality assessment

Using the Cochrane Risk of Bias Assessment Tool, two researchers independently assessed the quality of the included literature [10]. The assessment included seven domains: random sequence generation, allocation concealment, blinding of participants and personnel, blinding of outcome assessment, incomplete outcome data, selective reporting, and other sources of bias. Each field’s risk level is divided into three levels: high risk, unclear risk, and low risk. If there are discrepancies in the evaluations of two researchers, a discussion with a third researcher will be held to reach a consensus and resolve the discrepancies.

### Data extraction

Valuable information was obtained from the included randomized controlled trials, including: (1) The first author’s name and publication year of each randomized controlled trial. (2) Country and sample size. (3) Treatment drug and dosage. (4) Study outcomes, primarily assessed using the Ureteral Stent Symptom Questionnaire (USSQ). These variables were only analyzed when assessed by two or more studies.

### Compliance with ethics guidelines

This article is based on previously conducted research and does not contain any new studies on human participants or animals by any of the authors.

### Statistical analyses

We processed the extracted data using RevMan version 5.4 [11]. For continuous data, mean difference (MD) and 95% confidence interval (CI) were chosen for evaluation. For dichotomous data, relative risk (RR) and 95% CI were used for evaluation [12]. The chi-square test was used to assess heterogeneity among the studies. If I^2^ > 50%, it indicates significant heterogeneity, and a random-effects model is used. If I^2^ < 50%, it means that the heterogeneity is not significant, and a fixed-effects model is used. When p-value is less than 0.05, it indicates that the difference in outcome measures between the experimental and control groups is statistically significant.

## Results

### Study selection, search results, and characteristics of the trials

We followed the above search strategy and eventually retrieved 239 articles, 206 of which were excluded by eliminating duplicates and filtering titles and abstracts. Of the remaining 33 articles, 25 were excluded due to either not being found in the literature search or lacking valid data after reading the full articles, and eight studies were ultimately included to evaluate the efficacy and safety of tolterodine and α-adrenergic receptor blockers for the treatment of ureteral stent-related symptoms [8, 13–19]. The details, characteristics, and baseline summaries of the included studies are shown in Table 1.

**Table 1:** Basic characteristics of included studies.

Country	Experimental group (M)	Control group (C)	Outcomes	Sample size		Inclusion population	Study design
				M	C		
Tunisia	tolterodine (4 mg/day)	tamsulosin (0.4 mg/day)	IPSS/QoL/VAPS	25	25	All patients underwent ureteroscopic for ureteral distal calculi with indweling ureteral double-j stent.	The IPSS/Qol and VAPS were completed by each patient before stent insertion, at 1st day, 14th day and 30th day postoperatively.
India	tolterodine (4 mg/day)	tamsulosin (0.4 mg/day)	USSQ/VAPS	50	50	All patients above 18 years of age undergoing unilateral DJ stenting following aforementioned urological procedures.	To assess the stent-related morbidity, all patients completed validated USSQ, 1 week after the DJ stent insertion and at 4 weeks (stent removal).
Iran	tolterodine (4 mg/day)	tamsulosin (0.4 mg/day)	USSQ	43	40	All the patients we involved in study were underwent unilateral ureteral stenting for relieving upper urinary tract obstruction due to stone.	All of indices measured by USSQ for first and fourth weeks after drug consumption and the first week after double J stent removal (labeled as w1, w4, and w5, respectively).
Korea	tolterodine (4 mg/day)	alfuzosin(10mg/day)	USSQ	20	20	This study was unilateral placement of a Double-J ureteral stent after ureteral surgery, including ureteroscopy, percutaneous nephrolithotomy, and ureteroplasty.	All patients completed the validated USSQ 6 weeks after the stent insertion to assess the stent-related morbidity.
Iran	tolterodine (2 mg/day)	terazosin(4mg/day)	IPSS/QoL	23	23	Patients who were 18-55 years old with stone size ≤ 20 mm and underwent unilateral ureteral stenting entered in this trial.	Prior to stenting and at stent removal, the IPSS,QoL subscore and the VAS for Pain were determined.
Egypt	tolterodine (4 mg/day)	tamsulosin (0.4 mg/day)	USSQ/VAPS/QoL	38	40	Patients between 20 and 50 years old, who had been managed for a single ureteral stone less than 10 mm in their longest diameter, were included in the current randomized cohort study.	Pre-treatment evaluation was done followed by among-groups comparison after 14 days including USSQ,VAS and QOL.
India	tolterodine (4 mg/day)	tamsulosin (0.4 mg/day)	USSQ	20	20	Patients with symptomatic ureteric stones (less than 15mm) who were planned for ureteroscopic stone removal by ureterorenoscopy and unilateral double J stent placemen.	Every patient was evaluated with USSQ score at baseline after two weeks, four weeks and six weeks of treatment.

### Risk of bias

**Fig 2:**
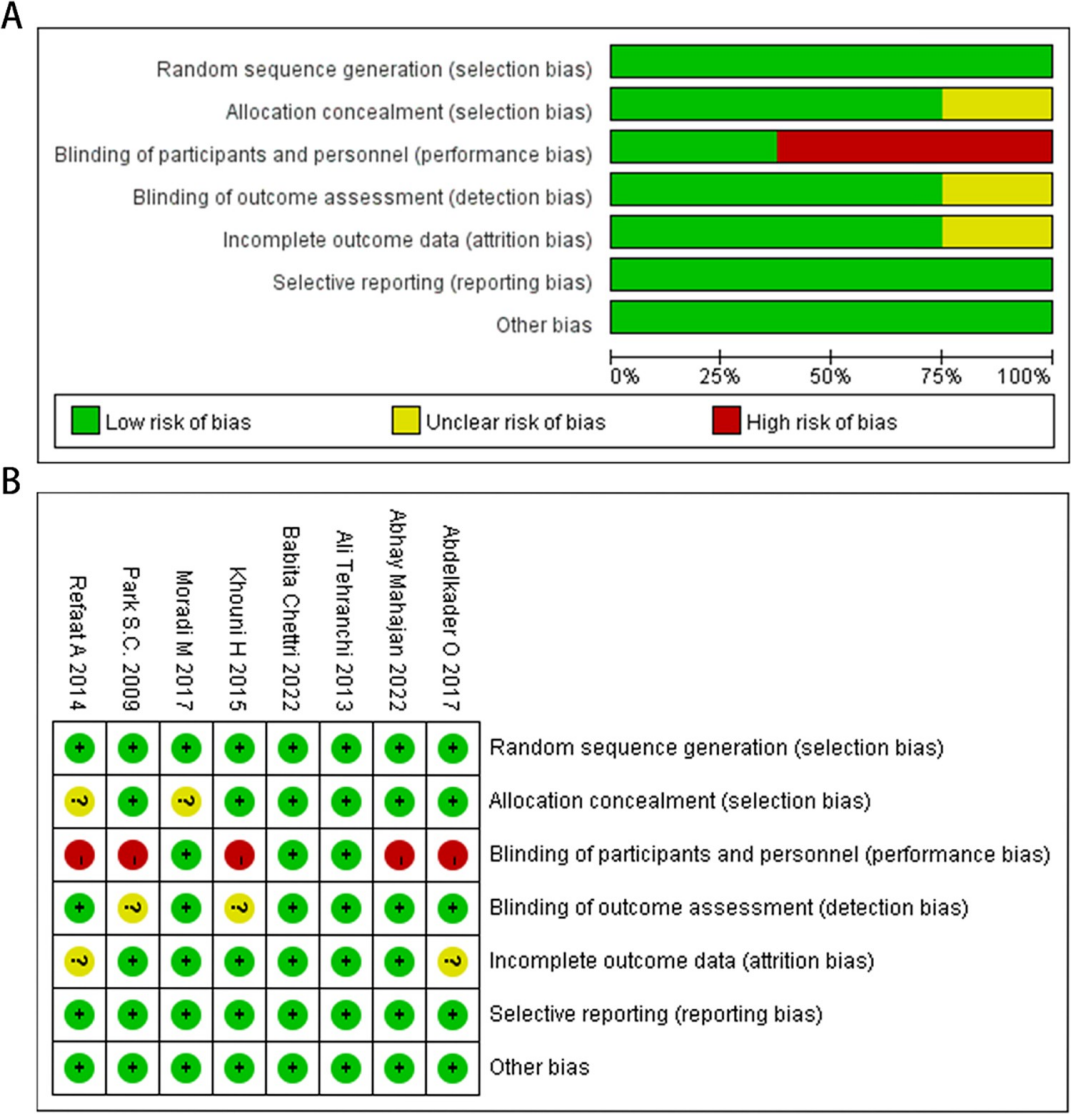
(A-B) Risk of bias summary.

This study is a clinical drug study. The 8 articles analyzed are all RCT articles, among which 3 are double-blind and complete in terms of complete data results and concealment of allocation scheme. Methods of quality assessment for each trial were reported using the Cochrane Bias Risk tool. The summary and chart of deviation risk are shown in Fig 2A and 2B.

### The therapeutic efficacy after two weeks

**Fig 3:**
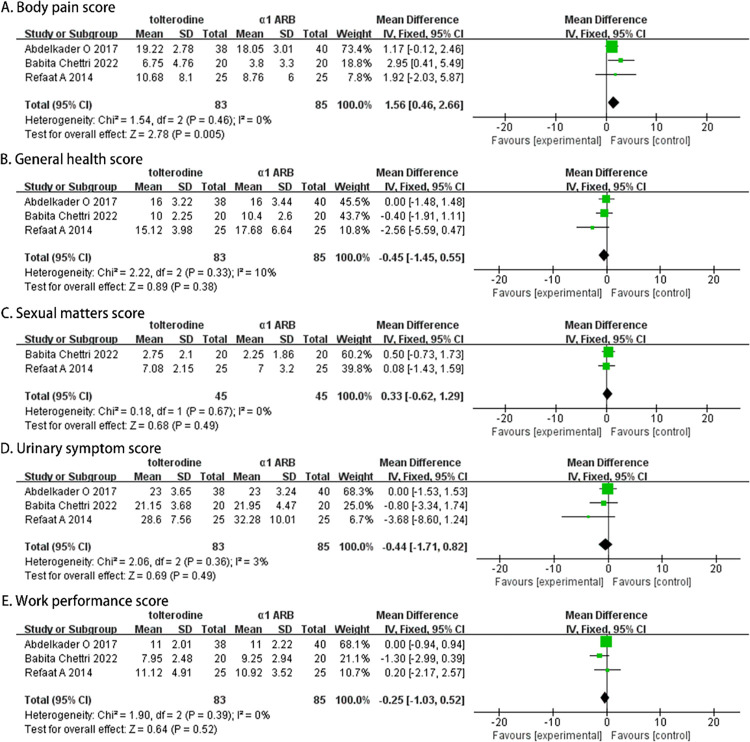
Forest plots comparing tolterodine with alpha-adrenergic blockers for Ureteral Stent Symptom Questionnaire (USSQ) (A) body pain score, (B) general health score, (C) sexual matters score, (D) urinary symptom score, and (E) work performance score at 2 weeks.

A total of 168 participants (83 in the α-adrenergic receptor blockers group and 85 in the Tolterodine group) were recruited in three studies, and their USSQ scores at week 2 were analyzed. The fixed-effects model revealed that Tolterodine significantly alleviated body pain symptoms (MD, 1.56; 95% CI [0.46, 2.66]; p = 0.005; Fig 3A). However, there were no statistically significant differences between the two groups in terms of improving urinary symptoms (MD, -0.44; 95% CI [-1.71, 0.82]; p = 0.49; Fig 3D), general health (MD, -0.45; 95% CI [-1.45, 0.55]; p = 0.38; Fig 3B), work performance (MD, -0.25; 95% CI [-1.03, 0.52]; p = 0.52; Fig 3E), and sexual performance (MD, 0.33; 95% CI [-0.62, 1.29]; p = 0.49; Fig 3C).

### The therapeutic efficacy after four weeks

**Fig 4:**
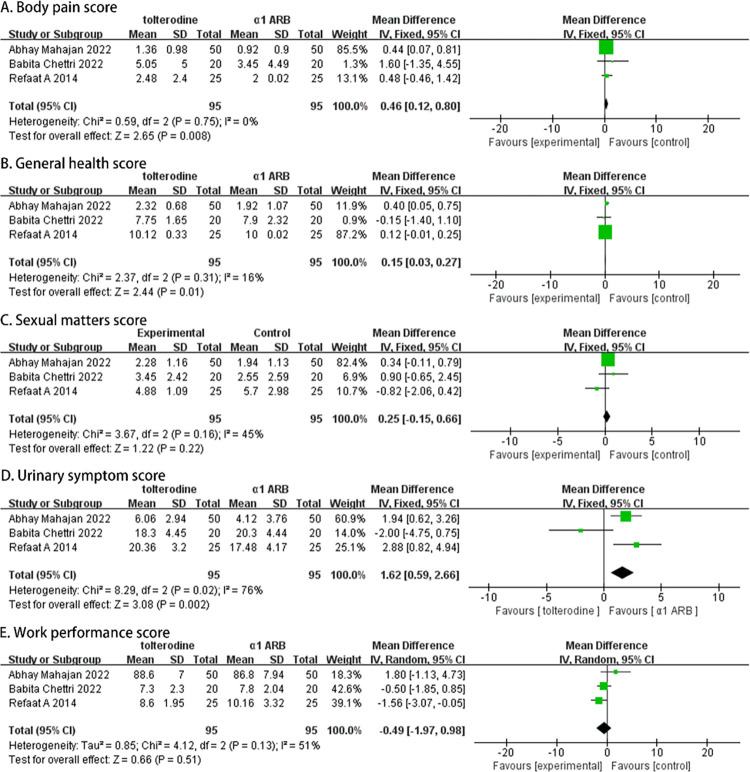
Forest plots comparing tolterodine with alpha-adrenergic blockers for Ureteral Stent Symptom Questionnaire (USSQ) (A) body pain score, (B) general health score, (C) sexual matters score, (D) urinary symptom score, and (E) work performance score at 4 weeks.

A total of 190 participants (95 in the α-adrenergic receptor blockers group and 95 in the Tolterodine group) were recruited in three studies, and their USSQ scores at week 4 were analyzed. Both random-effects and fixed-effects models were employed, and the results indicated that Tolterodine significantly alleviated body pain symptoms (MD, 0.46; 95% CI [0.12, 0.80]; p = 0.008; Fig 4A), general health (MD, 0.15; 95% CI [0.03, 0.27]; p = 0.01; Fig 4B), and urinary symptoms (MD, 1.62; 95% CI [0.59, 2.66]; p = 0.002; Fig 4D). However, there were no significant differences observed in the USSQ domain scores for work performance (MD, -0.49; 95% CI [-1.97, 0.98]; p = 0.51; Fig 4E) and sexual performance (MD, 0.25; 95% CI [-0.15, 0.66]; p = 0.22; Fig 4C).

### The therapeutic efficacy after six weeks

**Fig 5:**
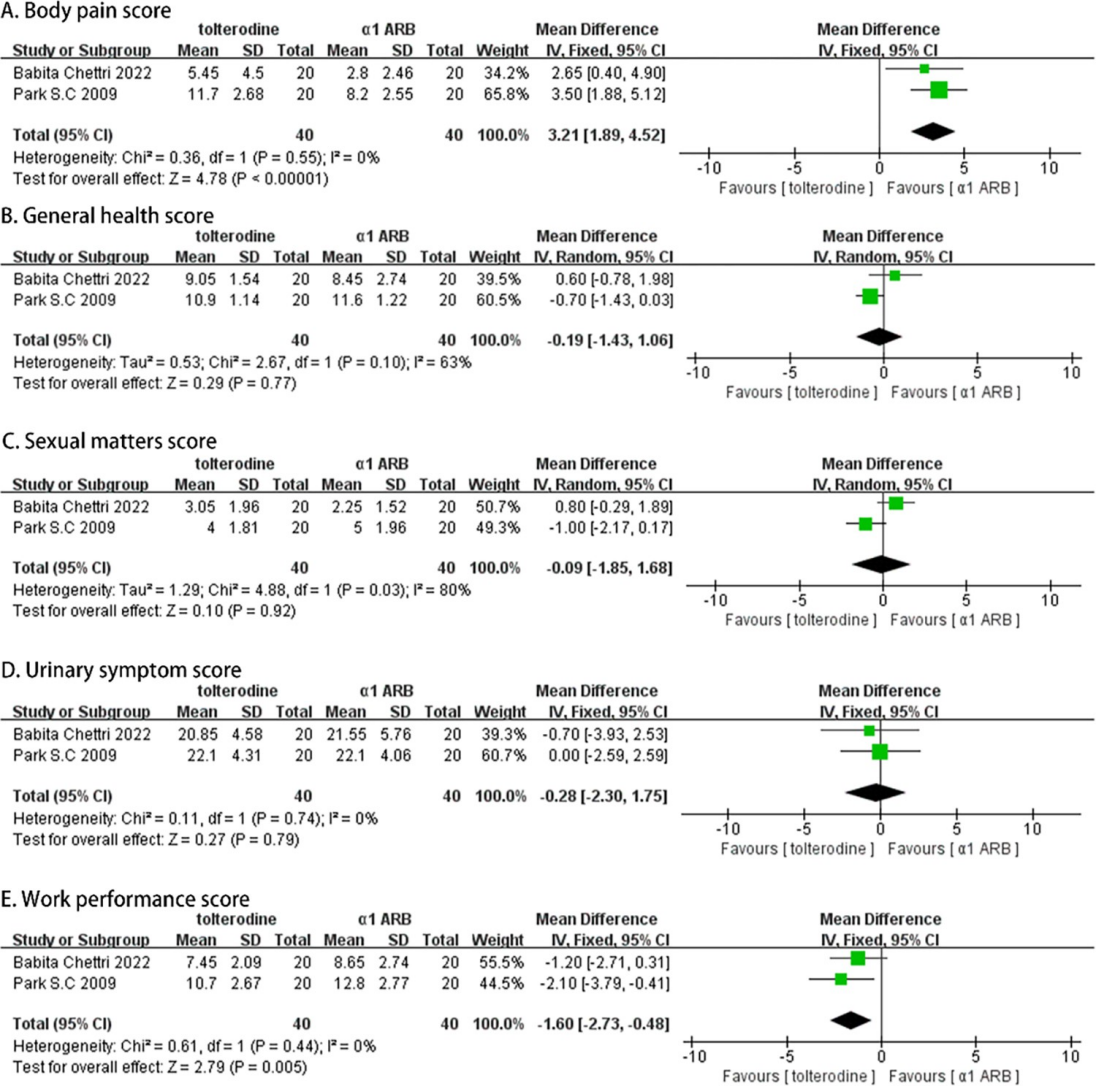
Forest plots comparing tolterodine with alpha-adrenergic blockers for Ureteral Stent Symptom Questionnaire (USSQ) (A) body pain score, (B) general health score, (C) sexual matters score, (D) urinary symptom score, and (E) work performance score at 6 weeks.

Two studies analyzed the USSQ scores at week 6, involving a total of 90 participants (45 in the α-adrenergic receptor blockers group and 45 in the Tolterodine group). Both random-effects and fixed-effects models were used, and the results showed that Tolterodine significantly alleviated body pain symptoms (MD, 3.21; 95% CI [1.89, 4.52]; p = 0.00001; Fig 5A), while α-adrenergic receptor blockers significantly improved work performance (MD, -1.60; 95% CI [-2.73, -0.48]; p = 0.005; Fig 5E). However, there were no significant differences observed in the USSQ domain scores for urinary symptoms (MD, -0.28; 95% CI [-2.30, 1.75]; p = 0.79; Fig 5D), general health (MD, -0.19; 95% CI [-1.43, 1.06]; p = 0.77; Fig 5B), and sexual performance (MD, -.0.09; 95% CI [-1.85, 1.68]; p = 0.92; Fig 5C).

### Postoperative complication

**Fig 6:**
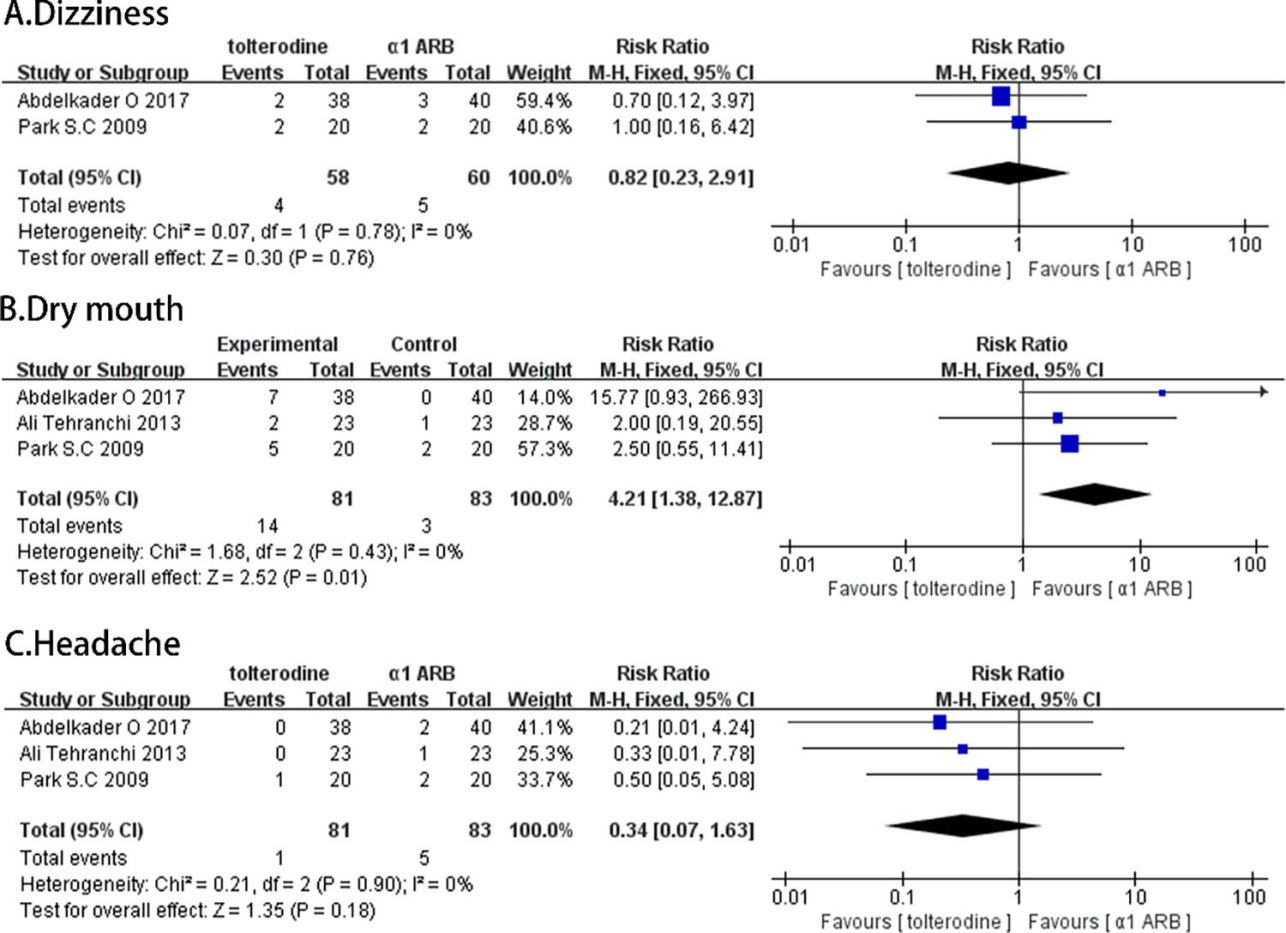
Forest plots comparing tolterodine with alpha-adrenergic blockers for (A) dizziness, (B) dry mouth, and (C) headache.

Three randomized controlled trials reported dry mouth changes in 164 patients. The random effects model showed that the incidence of dry mouth was significantly higher in patients receiving tolterodine treatment than in α-adrenergic receptor blockers (RR, 4.21; 95% CI [1.38, 12.87]; p = 0.01; Fig 6B). There was no significant difference in the incidence of dizziness (RR, 0.82; 95% CI [0.23, 2.91]; p = 0.76; Fig 6A) and headache (RR, 0.34; 95% CI [0.07, 1.63]; p = 0.18; Fig 6C).

## Discussion

The ureteral stent, initially introduced by Zimskind et al. in 1967, was originally designed as a straight tube [20]. However, due to the tendency of the straight stent to migrate or slip out, it was later optimized into a double “J” shape [21]. As a result, it is commonly referred to as a double-J stent. The extensively refined double-J stent has now emerged as an indispensable instrument in the realm of urological surgery. Despite the widespread utilization of ureteral stents in urological surgeries, they are associated with post-procedural urinary symptoms, discomfort, and diminished quality of life [22]. Joshi et al. indicate that following ureteral stent placement, approximately 80% of patients experience urinary system symptoms and pain, 58% exhibit decreased work performance, and 32% encounter sexual dysfunction [23].

Despite the increasing number of studies exploring the mechanisms underlying the occurrence of symptoms associated with ureteral stents, the pathophysiology of these symptoms still lacks a definitive explanation. Lang et al. proposed that stent-related pain and urinary symptoms may be the result of ureteral spasm or neurostimulation of the trigone region of the bladder, leading to involuntary contractions of the bladder [24]. Additionally, these symptoms may be further exacerbated by increased pressure transmitted to the renal pelvis during voiding, bladder ischemia, and spasms in the lower ureter and bladder [25, 26]. The placement of a stent may also exacerbate pre-existing subclinical detrusor overactivity and induce symptoms of bladder overactivity. In the past, we have attempted to improve the associated discomfort symptoms caused by stents through optimizing stent design. However, a recent study has indicated that new stents do not improve stent-related symptoms [27].

Alpha-adrenergic blockers are first-line medications for the treatment of lower urinary tract symptoms, while M receptor blockers are widely used in the treatment of bladder overactivity. Since the urinary symptoms associated with stent placement are similar to those caused by bladder overactivity and symptoms related to benign prostatic hyperplasia, these two medications have been used in clinical practice to treat stent-related symptoms [8]. Alpha-adrenergic blockers work by blocking alpha-adrenergic receptors, thereby reducing muscle tone in the ureter, bladder trigone, and prostatic urethra. This reduction in muscle tone helps to decrease resistance and pressure at the bladder outlet during urination, ultimately relieving lower back pain. Abdominal pain may be a result of ureteral spasms caused by the presence of a stent. Alpha-adrenergic blockers can help alleviate abdominal pain by reducing ureteral spasms and preventing bladder-ureter reflux [28]. Tamsulosin, an exquisitely selective α1 receptor antagonist, exerts its mechanism of action by inhibiting the α1 receptors on the smooth muscles of the prostate, bladder, and urethra, inducing relaxation in the urinary tract smooth muscles. The adverse reactions associated with tamsulosin encompass headaches, dizziness, muscular weakness, nasal bleeding, retrograde ejaculation, and floppy iris syndrome. The administration of anticholinergic agents can effectively alleviate bladder overactivity and contractions by antagonizing muscarinic receptors, thereby reducing urinary symptoms [29]. Numerous studies have been conducted to validate its therapeutic efficacy, yielding definitive outcomes. Tolterodine, in particular, stands as a frequently employed anticholinergic agent in clinical practice.

In the past, we commonly utilized indicators such as IPSS, QoL, and VAPS to assess symptoms related to stent placement. However, these assessments lack specificity when it comes to evaluating symptoms specifically associated with ureteral stent implantation and have yet to be accurately validated. USSQ, on the other hand, is a validated questionnaire composed of six domains: urinary symptoms, body pain, general health, work performance, sexual issues, and other concerns, designed to assess symptoms related to stent placement [30]. Compared to validation indicators like IPSS, this questionnaire has been thoroughly validated in various language versions and exhibits optimal reliability [31, 32].

Previous studies have confirmed the effectiveness of α-adrenergic receptor blockers and tolterodine in alleviating symptoms associated with ureteral stents. However, there are differing opinions regarding the comparative efficacy of these two drugs in treating discomfort related to ureteral stents. Wang et al. compared the efficacy of the antimuscarinic drug solifenacin and the α-adrenergic blocker tamsulosin as monotherapy for the treatment of ureteral stent-related symptoms [33]. The results showed that solifenacin was only superior to tamsulosin in improving sexual function, with no significant differences in other aspects. Jian et al. further investigated the efficacy of solifenacin in relieving ureteral stent-related symptoms when compared to the α-adrenergic blockers tamsulosin or alfuzosin as single agents [34]. They found that solifenacin was more effective in relieving body pain than tamsulosin or alfuzosin. In 2019, Gao et al. conducted a meta-analysis of randomized controlled trials comparing various antimuscarinic drugs, including tolterodine, with different α-adrenergic blockers in the treatment of ureteral stent-related symptoms [35]. The results showed that there was no significant difference in efficacy between antimuscarinic drugs and α-adrenergic blockers. However, the previous relevant meta-analysis did not separately analyze the efficacy differences in alleviating symptoms related to ureteral stents between tolterodine and alpha-adrenergic receptor blockers (mainly tamsulosin). Additionally, some studies included in the analysis had a small sample size or only analyzed certain subscales of the USSQ.

To the best of our knowledge, this is the first meta-analysis utilizing USSQ scores to comprehensively evaluate the efficacy and complications of α-adrenergic receptor blockers and tolterodine as monotherapy for relieving symptoms associated with ureteral stents. Our study further revealed that compared to tolterodine, α-adrenergic receptor blockers demonstrated statistically significant advantages in alleviating bodily pain, while tolterodine exhibited superior effects in enhancing work capacity. Previous studies have found that tolterodine, as an antimuscarinic drug, reduces the frequency and pressure of non-voiding contractions (NVCs) in bladder outlet obstruction (BOO) rats [36]. Sugiyama et al. found that silodosin affects the number of NVCs, while imidafenacin affects both the number and average amplitude of NVCs [37]. It can be seen that antimuscarinic drugs are more effective in relieving and treating symptoms such as urinary frequency caused by irregular bladder contractions than α-adrenergic receptor blockers. Symptoms such as urinary frequency and urgency are mainly caused by the stimulation of the bladder trigone mucosa by ureteral stent implantation. Tolterodine acts on the M receptor in the bladder trigone area, reducing involuntary bladder contractions, decreasing urinary frequency, and thus increasing efficiency. Our meta-analysis found that at week 4, compared to tolterodine, α-adrenergic receptor blockers improved lower urinary tract symptoms, but there was no significant difference between the two at weeks 2 and 6. We speculate that the significant improvement in lower urinary tract symptoms at week 4 compared to weeks 2 and 6 may be due to bias caused by the different studies included in each time subgroup. It is also possible that with prolonged use of α-adrenergic receptor blockers, the therapeutic effect may gradually surpass that of tolterodine. However, the 6-week group had fewer studies included compared to the 2-week and 4-week groups, and the inclusion criteria varied, potentially introducing bias. We hope for more long-term studies in the future to further explore whether there is a difference in the efficacy of tolterodine compared to α-adrenergic receptor blockers in treating symptoms related to ureteral stent placement. In terms of complications, because tolterodine blocks the M receptor, the incidence of dry mouth is higher compared to α-adrenergic receptor blockers.

Our meta-analysis possesses several strengths. Firstly, the studies analyzed in our research were all randomized controlled trials, with some being double-blind randomized controlled experiments, indicating a low risk of bias. Secondly, to the best of our knowledge, there have been no reports attempting to comprehensively analyze the effectiveness and safety of tolterodine in combination with α-blockers for the treatment of symptoms related to ureteral stent placement. Our study provides guidance for the clinical application of tolterodine and α-blockers in managing symptoms associated with ureteral stents. However, this study also has some limitations. Firstly, various clinical factors and any underlying ureteral conditions can influence the results, and different patient characteristics may also impact the overall outcomes. Secondly, due to data limitations, our study only compared the efficacy of tamsulosin, an α-adrenergic receptor blocker, with tolterodine. Thirdly, although the included randomized controlled trials were of high quality, they were relatively few, and the sample sizes were small. Further research is needed to expand the comparative analysis of the efficacy of these two medications.

## Conclusion

In summary, α-adrenergic receptor blockers have shown superiority over tolterodine in alleviating bodily pain caused by ureteral stents, while tolterodine has demonstrated better effects in enhancing work capacity compared to α-adrenergic receptor blockers. Additionally, the incidence of dry mouth is higher with the use of tolterodine compared to α-adrenergic receptor blockers. In the future, it is imperative to conduct more large-scale, long-term follow-up, and high-quality randomized controlled trials in this field to provide stronger evidence and further evaluate and validate these findings.

## Supporting information

S1 Checklist PRISMA 2020 checklist. (DOCX)

S1 Data Minimal dataset underlying the results. (XLSX)
